# Past local government health spending was not correlated with COVID-19 control in US counties

**DOI:** 10.1016/j.ssmph.2022.101027

**Published:** 2022-01-18

**Authors:** Sneha Lamba, Carrie Wolfson, Carolina Cardona, Y. Natalia Alfonso, Alison Gemmill, Beth Resnick, Jonathon P. Leider, J. Mac McCullough, David Bishai

**Affiliations:** a Johns Hopkins Bloomberg School of Public Health, USA; b University of Minnesota School of Public Health, USA; c Arizona State University, USA

**Keywords:** LHD, Local Health Department, PH, Public Health, Public health spending, COVID-19 control, Local health departments, Foundational capabilities

## Abstract

**Context:**

Wide variation in state and county health spending prior to 2020 enables tests of whether historically better state and locally funded counties achieved faster control over COVID-19 in the first 6 months of the pandemic in the Unites States prior to federal supplemental funding.

**Objective:**

We used time-to-event and generalized linear models to examine the association between pre-pandemic state-level public health spending, county-level non-hospital health spending, and effective COVID-19 control at the county level. We include 2,775 counties that reported 10 or more COVID-19 cases between January 22, 2020, and July 19, 2020, in the analysis.

**Main outcome measure:**

Control of COVID-19 was defined by: (i) elapsed time in days between the 10th case and the day of peak incidence of a county's local epidemic, among counties that bent their case curves, and (ii) doubling time of case counts within the first 30 days of a county's local epidemic for all counties that reported 10 or more cases.

**Results:**

Only 26% of eligible counties had bent their case curve in the first 6 months of the pandemic. Government health spending at the county level was not associated with better COVID-19 control in terms of either a shorter time to peak in survival analyses, or doubling time in generalized linear models. State-level public spending on hazard preparation and response was associated with a shorter time to peak among counties that were able to bend their case incidence curves.

**Conclusions:**

Increasing resource availability for public health in local jurisdictions without thoughtful attention to bolstering the foundational capabilities inside health departments is unlikely to be sufficient to prepare the country for future outbreaks or other public health emergencies.

## Highlights

• Substantial variation in county and state health spending in the US before the COVID-19 pandemic.
• County level non-hospital health spending before pandemic not associated with early control over COVID-19.
• State level spending on hazard preparation is associated with early control over COVID-19 in some model specifications.
• Results offer no support for pouring funds into public health system to prepare for future outbreaks.

## 1 Introduction

The failure to control the first wave of the COVID-19 pandemic in most parts of the US provokes important questions about the capability of local public health (PH) departments to handle a public health crisis (Maani & Galea, 2020). To the extent that financial investments in public health (PH) capacity ahead of a crisis help mitigate its severity, pre-pandemic spending by local health departments (LHDs) theoretically ought to have helped control the early spread of COVID-19 — if annual spending included meaningful amounts of investment in the ‘foundational capabilities’ in PH. Foundational capabilities include epidemiological assessment, emergency response, communications, administration, and community partnership (RESOLVE – Public Health Leadership Forum, 2014; Kalyanaraman & Fraser, 2021; Fox, 2020). Had past local government health spending included capacity-building it would have given health departments personnel and resources, to allow them to surge into needed roles in surveillance, communications, procurement, accessing state and federal funds, human resources, and community coordination. Counties lacking these foundational capabilities would have to play catch-up during a crisis akin to “patching a roof during a hurricane”.

If capability is a product of past financial investments, then it is important to ask whether county governments with higher levels of pre-pandemic health spending were better able to control the early emergence of the epidemic. LHDs have been at the forefront of epidemic management and rallying community efforts for emergency preparedness in the past. The COVID-19 pandemic is no exception (Ravenhall et al., 2021)^,^ (Schoch-Spana et al., 2018). If COVID-19 breached the defenses of well-funded and poorly funded LHDs with equal intensity, then it would trigger policymakers to focus on more fundamental restructuring of PH practice in the US. Testing whether state and county governments’ past spending on health hastened control of the outbreak would shed light on the adequacy of financing alone to shore up the US PH infrastructure.

This paper uses systematic data on government health spending at the county level, and PH spending at the state level, prior to the start of the pandemic to address this question. Data on county-level health spending can only be broadly categorized into spending on hospitals and spending “not on hospitals”. Here spending “not on hospitals” includes spending on PH departments as well as outpatient clinics and emergency medical services, etc. On the other hand, state government health spending data can be categorized much more finely to examine spending on hazard preparedness and communicable disease control.

A large body of literature has investigated the association between local government expenditures on PH and population health outcomes at the LHD level (Cardona et al., 2021), (McCullough & Leider, 2016). Longitudinal studies by Erwin et al., 2011, Erwin et al., 2012 find positive associations between LHD spending aggregated to the state level and state-level infectious disease mortality (Erwin et al., 2011)^,^ (Erwin et al., 2012). Similarly, Mays and Smith (2011) find a positive association between LHD spending, and the outcomes of infant mortality and deaths attributable to cardiovascular disease, diabetes, and cancer (Mays & Smith, 2011). A systematic review that includes all studies carried out between 1985 and 2012, which have examined the relationship between PH spending and population health outcomes found that increases in PH spending at the state, or local county level were largely associated with improved population health outcomes. However, the pathways through which spending improved health was not well understood (Singh, 2014). Conceptual models developed by Handler et al. (2001) and refined by Meit et al. (2012) suggest that the organizational capacity within the PH system plays a key role in their ability to improve internal processes, and performance, and ultimately population health outcomes (Handler et al., 2001; Meyer et al., 2012; Singh, 2014). Financial resources are an important component of the organization capacity of LHDs, and previous studies have demonstrated how financial resources are associated with improved performance (Hyde & Shortell, 2012). To the best of our knowledge no prior U.S. study has examined how PH spending at the LHD and state level is associated with the ability to manage emergencies such as the COVID-19 pandemic. A paper that examines this question using data from the United Kingdom showed no effect of local PH spending on the pace of control of COVID-19 (Acharya et al., 2021).

The governmental PH system in the U.S. is composed of a complex network of organizations with varying degrees of collaboration at the national, state, and local levels (Hyde & Shortell, 2012). LHDs are governed differently in centralized, decentralized, and mixed or shared states. In centralized states, LHDs are operated by state health agencies (SHAs). In decentralized states (or mixed, and shared states) LHDs retain more authority and responsibility for the delivery of PH services (Leider et al., 2018). However, even in centralized states it is not uncommon for LHDs to have some authority over the allocation of funds (Meit et al., 2012). Similarly, in decentralized states, states often contribute financial resources to LHDs that are tied to guidelines on how they can be spent (Meit et al., 2012). We hypothesize that across the different PH systems of the U.S., LHDs with higher health spending per capita would have had access to more staff with skills to detect and track the earliest outbreaks. Greater resources would be associated with surge capacity and partnerships with the community in order to build community trust, cooperation, and the ability to execute the policies required to achieve compliance with quarantine, tracking, and testing (Singh, 2014)^,^ (Scutchfield et al., 2009). We hypothesize that these pre-existing assets could lead to greater and earlier local level success in sounding well-timed alarms to the community and local leaders, who could then mobilize rapid and effective non-pharmaceutical intervention (NPI) responses to COVID-19 ^18,19^. In contrast, a null relationship between pre-pandemic government spending at the county level, and early ability to control COVID-19 would trigger consideration of barriers that block LHDs from building their foundational capabilities out of their annual fiscal appropriations.

Our study design is based on noting that prior to the COVID-19 pandemic there was substantial variation in the government health spending of counties in the US (Singh et al., 2020). Even though pre-pandemic LHD budgets would have been mostly sequestered in earmarked categorical grants unrelated to communicable disease, our hypothesis assumes that all LHDs have had to redeploy categorical staff to new roles in the early COVID-19 efforts. We test whether the health departments who had larger total pre-pandemic budgets would have built up staff with foundational capabilities in PH that would lead to better pandemic control (Leider et al., 2018).

We measure the control of COVID-19 in terms of counties' ability to bend COVID-19 case incidence curves in the first 180 days of the pandemic. We restricted the analysis to the first 6-months of response, not because PH practices after July 2020 were unimportant. On the contrary, the release of Coronavirus Aid, Relief, and Economic Security (CARES) Act funds to counties and their LHDs in the second part of 2020 substantially overwhelmed prior PH investments in magnitude. This increase in funding also influenced the multiple resurgent epidemic waves in late 2020, and then in 2021, overshadowing the pandemic's first few months in terms of devastation. We argue that the adequacy of pre-COVID level spending on PH preparedness can only be properly tested in the early months of an epidemic before reinforcements and supplemental funding arrived. Relief funding that arrived in mid-2020 was allocated to health entities in accordance with a formula that accounts for revenue, location, insurance rates, and COVID-19 hospitalization rates. As a result, we will no longer be able to test the adequacy of pre-COVID PH spending as a protective measure against the first wave of COVID-19 in the latter half of the year (Vahidy et al., 2020). Supplemental COVID-19 response resources were allocated both in proportion to epidemiological need and ability to request and secure outside assistance (Department of the Treasury Office of Inspector General (OIG), 2020)^,^ (Dirlikov et al., 2020). Given this funding allocation, the geographical pattern of CARES Act spending correlated with greater financial and epidemiological need which would invalidate an attempt to test whether the pre-COVID 2020 government health spending levels were associated with more rapid success in disease control. By late spring and summer of 2020, reinforcements of CARES Act funding and CDC personnel deployments would start to weaken any relationship between past spending and contemporary success in controlling COVID-19 in its mid and late 2020 manifestation (Department of the Treasury Office of Inspector General (OIG), 2020).

## 2 Methods

### 2.1 Data sources

We compiled data for this study from a variety of sources, which are listed in Supplemental Appendix Table 2. We only provide details on the key independent and dependent variables here. Daily COVID-19 cumulative cases were obtained from the *New York Times*. Annual county and state level public expenditure data were extracted from the US Census Bureau's local and state finance files for the years 2015-2017 at the county level, and 2016–2018 at the state level. The US Census Bureau's division of state finance provides annual data on state level expenditures while data on county level expenditures is obtained from the Census of Local Governments, conducted by the US Census Bureau every five years. Details about coding census data on state health expenditure into functional categories and local quinquennial spending estimates into annual spending estimates has been described elsewhere (McCullough & Leider, 2016)^,^ (Leider et al., 2018)^,^ (Leider et al., 2016)^,^ (Resnick et al., 2017).

**Table 1:** Effect of county-level spending - Estimated odds ratios from AFT models with time to peak as dependent variable.

	Spending only	Spending + Testing + Demographic	Spending + Testing + Demographic + Income	Spending + Testing + Demographic + Income + Health	Spending + Testing + Demographic + Income + Health + Temperature	Spending + Testing + Demographic + Income + Health + Temperature + Political
	(1)	(2)	(3)	(4)	(5)	(6)
	Model 1	Model 2	Model 3	Model 4	Model 5	Model 6
Ln(County Non-Hospital Health Spending Per Capita)	1.043	0.992	0.990	0.993	0.993	0.993
	[0.0584]	[0.0574]	[0.0571]	[0.0569]	[0.0559]	[0.0561]
Ln(County Revenue Per Capita)	0.449***	0.638*	0.664	0.715	0.751	0.756
	[0.107]	[0.162]	[0.171]	[0.185]	[0.194]	[0.198]
Ln(County Hospital Spending Per Capita)	0.983	0.986	0.986	0.981	0.980	0.980
	[0.0178]	[0.0186]	[0.0186]	[0.0187]	[0.0186]	[0.0187]
Ln(County Public Welfare Spending Per Capita)	0.983	0.988	0.992	1.002	1.017	1.018
	[0.0490]	[0.0509]	[0.0518]	[0.0511]	[0.0509]	[0.0511]

No. of Records	147,346	147,346	147,346	147,346	147,346	147,346
No. of Groups	48	48	48	48	48	48
AIC	2846	2721	2723	2718	2712	2714
No. of Subjects	1957	1957	1957	1957	1957	1957
Time at Risk	151676	151676	151676	151676	151676	151676

seEform in brackets. Spending coefficients correspond to natural log of 1 + actual spending to avoid taking log of zero.

***p < 0.01, **p < 0.05, *p < 0.1.

Number of Records (Observations) is number of days a county has been observed prior to failure or right censoring. Each subject in our analysis has been observed for a minimum of 1 time and a maximum of 144 times with subjects being observed for a median of 86 times. The varying times that each subject has been observed is because a subject (or a county) enters the analysis only when it has had more than 10 cases of COVID-19 and leaves either when it has experienced the failure event (i.e. bent its case curve) or it was right censored (never bent its case curve).

Number of Groups is the number of US states. We assumed shared frailty at the state level to adjust for the non-independence of counties sharing exposure to state level COVID-19 control policies and PH spending policies.

Number of Subjects is the number of counties that had no missing values for any of the independent variables (including those that were right censored).

**Table 2:** Effect of state-level spending - Estimated odds ratios from AFT models with time to peak as dependent variable.

	Spending only	Spending + Testing + Demographic	Spending + Testing + Demographic + Income	Spending + Testing + Demographic + Income + Health	Spending + Testing + Demographic + Income + Health + Temperature	Spending + Testing + Demographic + Income + Health + Temperature + Political
	(1)	(2)	(3)	(4)	(5)	(6)
	Model 1	Model 2	Model 3	Model 4	Model 5	Model 6
Ln(State Per Capita Spending - Total)	0.701	0.716	0.721	0.917	0.915	0.911
	[0.155]	[0.176]	[0.185]	[0.216]	[0.192]	[0.192]
Ln(State Per Capita Spending - Hazard Preparation and Response)	0.593**	0.517***	0.512***	0.542***	0.600**	0.600**
	[0.136]	[0.120]	[0.123]	[0.121]	[0.122]	[0.122]
Ln(State Per Capita Spending - Communicable Disease Control)	1.042	0.973	0.978	1.018	0.934	0.936
	[0.149]	[0.164]	[0.173]	[0.149]	[0.119]	[0.119]

No. of Records	143,877	143,877	143,877	143,877	143,877	143,877
No. of Groups	47	47	47	47	47	47
AIC	2831	2693	2695	2689	2683	2685
No. of Subjects	1919	1919	1919	1919	1919	1919
Time at Risk	148132	148132	148132	148132	148132	148132

Spending coefficients correspond to natural log of 1 + actual spending to avoid taking log of zero.

seEform in brackets.

***p < 0.01, **p < 0.05, *p < 0.1.

Number of Records (Observations) is number of days a county has been observed prior to failure or right censoring. Each subject in our analysis has been observed for a minimum of 1 time and a maximum of 144 times with subjects being observed for a median of 86 times. The varying times that each subject has been observed is because a subject (or a county) enters the analysis only when it has had more than 10 cases of COVID-19 and leaves either when it has experienced the failure event (i.e. bent its case curve) or it was right censored (never bent its case curve).

Number of Groups is the number of US states. We assumed shared frailty at the state level to adjust for the non-independence of counties sharing exposure to state level COVID-19 control policies and PH spending policies.

Number of Subjects is the number of counties that had no missing values for any of the independent variables (including those that were right censored).

### 2.2 Quantifying COVID-19 control at the county level

We restricted our analysis to COVID-19 cases at the county level reported between January 22, 2020 and July 19, 2020 (the first 180 days of the pandemic). We considered only those counties that reported 10 or more cases within the period of our analysis. If a county never had more than 10 cases, the time to bend a curve was regarded as unmeaningful. Cases per 100,000 people measured daily were smoothed using a locally weighted regression of daily cases on the number of days. The smoothed curves were then used to estimate first and second derivatives to capture the slope, and rate of change of the slope of the epidemic curve of each county. A county was categorized as having “bent”^1^ its case incidence curve if there existed a transition from a rising to a falling first derivative of the smoothed incident case curves (our “bending” definition). We defined the incidence peak as the point in the smoothed incidence curve where the slope was equal to zero.

**Fig. 1:**
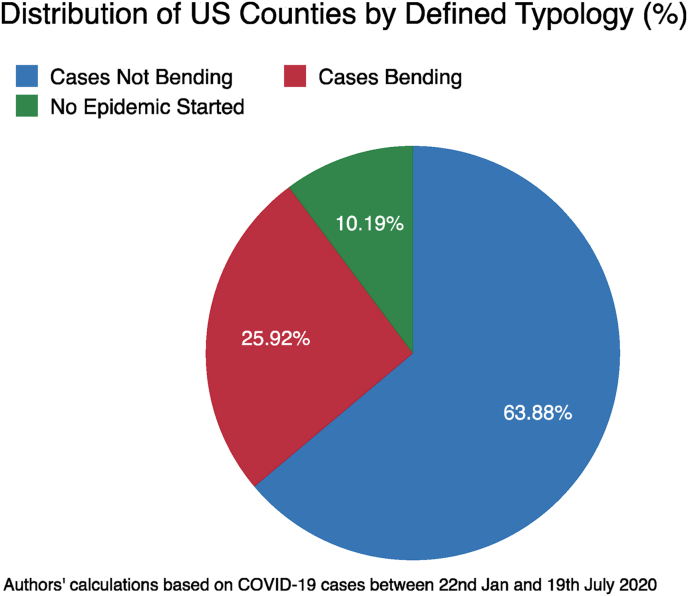
Distribution of US counties by defined typology (%).

By mid-July, many US counties were starting to have recurrent upward trends, suggesting they were due to have a second wave. We altered our curve bending definition to account for a second wave in these counties. A county with a potential second wave was defined as having successfully bent its COVID-19 curve by July 19, 2020 if it was able to drop down to an incidence level that was half of the incidence registered on the peak day, and if incidence had grown to no more than three quarters of prior peak incidence after July 19. Finally, we created a COVID-19 typology to classify counties into three groups: (i) cases bending; (ii) cases not bending; and (iii) no epidemic. For quality control, a team of student volunteers visually inspected county level case incidence curves, and denoted whether each county was classified correctly using the definition outlined above. The students found all counties were correctly classified. By July 19, 2020, only 26% of counties (N = 801) had successfully “bent their case curve” using our definition, 64% (N = 1974) had not bent their curves, and 10% (N = 315) had no epidemic (See Fig. 1).

### 2.3 Measures and variables

#### 2.3.1 Dependent variables

##### 2.3.1.1 Time to peak incidence

Time to peak case incidence measures the speed with which a county was able to slow the spread of a local COVID-19 outbreak. Shorter time to peak incidence signifies more success in outbreak control. We calculated this duration as time between the 10th case and the day an incidence curve bends based on the bending definition we outlined above.

##### 2.3.1.2 Doubling times in the first 30 days of the start of the epidemic

Considering that nearly 64% of US counties would be right censored in analyses that consider time to peak as the dependent variable, our analyses might fail to differentiate relative success among censored counties. To address this bias, we also computed the doubling time (DT) of case counts within the first 30 days of a county's local epidemic for all counties with at least 10 cases. The epidemiological rationale for restricting DT to the first 30 days was to stabilize the estimates of DT because the exponential growth phase of most epidemic curves would often begin to flatten after 30 days.^2^ The analytical rationale for focusing on DT only in the first 30 days of the pandemic was to maximize the relevance of the historical measures of local governmental health spending. As noted earlier throughout the second half of 2020 many counties obtained emergency funding that would augment and supersede indicators of historical staffing and capability. Random bad luck, like the docking of a cruise ship carrying infected persons or a super-spreader event, could have challenged some counties more severely in the first 30 days of their epidemic. However, if past local governmental health spending is to be regarded as a safeguard against outbreaks going out of control, the efficacy of this spending must be tested for effectiveness against all contingencies of bad luck. Effective LHDs in other countries did indeed cope with cruise ships and super-spreader events in 2020 (Kang, 2020; Nakazawa et al., 2020).

#### 2.3.2 Independent variables

##### 2.3.2.1 Health spending variables at county level

Our study examined three primary independent variables of interest related to county government health spending: non-hospital health spending, hospital health spending, and public welfare. We focus on non-hospital health spending because it is most relevant to the hypothesis that prior local government spending can improve control in the early months of a major epidemic. First, per capita county-level non-hospital health (NHH) consists of provision of services other than hospital care for the conservation and improvement of health and financial support of other government health programs; non-hospital health includes PH, public clinics, behavioral health spending, and disability-related clinical care (termed function 32 by the US Census bureau). Funding for local health departments would be included in NHH, as would funding for non-public health agencies, programs, and priorities. Second, per capita county-level public hospital spending consists of expenditures related to government's own hospitals, expenditures for the provision of care in other public hospitals, and direct payments for construction and acquisition of hospitals. Third, county-level public welfare spending consists of spending on all classes of welfare programs, including direct benefit transfers and administrative programs. These three categories of county spending are cross-correlated due to fluctuations in the local economy and political economy, so our models always include all three types of county spending, as well as county revenue per capita.

##### 2.3.2.2 Public health spending variables at state level

Total state per capita public health (PH) spending data offered a more detailed breakdown of sub-categories of spending than county spending did. The State Health Expenditure Dataset (SHED) from which the state level PH expenditures were drawn breaks down spending on categories of foundational capabilities (hazard preparedness and response, communications, policy development, assessment, community partnerships), and foundational areas (environmental health, chronic diseases, injury prevention, maternal and child health, access linkages, communicable diseases). Among these spending categories we pre-specified a focus on (i) Hazard Preparedness and Response (HPR), and (ii) Communicable Disease Control (CoDC) specifically owing to the high relevance of these two specific spending categories for outbreak preparedness (McCullough & Leider, 2016)^,^ (Leider et al., 2018)^,^ (Leider et al., 2016)^,^ (Resnick et al., 2017). However, PH spending on HPR, and CoDC, like other items in state public health budgets, rise and fall with politics and business cycles. Spending on any given sub-category of PH will thus be correlated with total state PH expenditure. Models of the impact of state spending on a sub-category like HPR or CoDC included a control variable for Total State Per Capita Spending. This avoided confounding bias which would attribute effects of a state's overall economic prosperity to one small sub-category of state spending. We specifically chose not to include multiple other state PH spending categories such as community partnerships or administration which would be collinear with spending on HPR and CoDC, but potentially less relevant to COVID control. Nevertheless, confounding bias of other omitted public spending or policy variables is still possible.

Expenditure data were inflation adjusted to 2018 USD (Leider et al., 2018)^,^ (Leider et al., 2016). We interpolated annual expenditure values for the year 2019 using linear regressions of the three most recent years of data: 2015–2017 for county and 2016–2018 for state data.

##### 2.3.2.3 Control variables

A key challenge to attributing positive health outcomes to county- or state-level spending is confounding. Counties and states that have high spending in the six categories constituting our primary predictors of interest may simply be those that are relatively disadvantaged or advantaged along several socio-economic determinants of health. Therefore, while our key hypothesis is to estimate the association between spending and COVID-19 response, we included individual, community, and health system-level risk factors to COVID-19 that have been identified by the emerging scientific literature on the pandemic as control variables in our study. The full list of control variables is detailed in Supplementary Table 1. Further, as noted above, the functional and administrative relationships between the state and local PH agencies may determine revenues and spending at the county and state level. We include state level control variables in our analysis to control for confounding at this level, which precludes the need to include state fixed effects.

Current research has highlighted how individual-level factors like age, pre-existing conditions, obesity, and smoking influence COVID-19 susceptibility and survival (Clark et al., 2020; Popkin et al., 2020; Reddy et al., 2021). Studies have shown how poverty and job insecurity are inextricably linked with people's ability to shelter-in-place or work-from-home (Wright et al., 2020). It is now widely acknowledged that Black, Indigenous, and People of Color (BIPOC) have borne a disproportionate burden of COVID-19 owing to structural and socio-economic determinants of health (Gelaye et al., 2020)^,^ (Chin et al., 2020). Any list of confounders cannot claim to include all possible socio-economic characteristics, underlying health conditions, and community as well as health system capacities as controls. Nonetheless, we have endeavored to include a wide set of controls informed by research on risk factors for COVID-19.

To test our hypothesis, models regressed COVID outcomes on state and local spending variables using separate specifications. We tested diverse sets of variables to control for COVID testing rates per capita, sociodemographic characteristics, nurses and doctors per capita, population general health indicators, weather temperatures, and 2016 presidential voting to model in a block-wise fashion the robustness of effects with different sets of confounders. (See Supplemental Appendix Table 1, Table 2). Control variables that had skewed distributions were included after log transformations. We added 1 prior to log transforming variables to avoid converting valid zeroes into missing values. We also estimated models with and without outliers. We only display and discuss models that have outliers removed. Models with outliers retained confirm the same results presented here.

### 2.4 Survival analysis

We used time to event models to estimate the impact of county and state spending on the time to peak incidence of local COVID-19. The variations in time to reach the peak are hypothesized to be affected by variations in regional factors at the county level through an accelerated failure time (AFT) model with time to peak as the variable to be explained by fixed time explanatory variables and a specified error term. Covariates act multiplicatively on the outcome of survival time. AFT models are parametric and require specification of a distribution for the baseline hazard function. The Weibull was selected as the best fit baseline hazard function based on the Akaike information criterion (AIC) that was used to compare each candidate distribution (Lee & Wang, 2003). AFT models allow for shared frailty to adjust for the non-independence of counties sharing exposure to state level COVID-19 control policies and governmental health spending policies. Shared frailty by state was assumed to be inverse Gaussian distributed.

### 2.5 Generalized linear models

We used generalized linear models (GLM) with logged dependent variables to estimate the association between past governmental spending at the county and state levels, and doubling time (DT) of incidence in the first 30 days of the pandemic among all counties who started their epidemic. We similarly controlled for the control variables described above and listed in Supplementary Table 1.

### 2.6 Checking for spatial autocorrelation

The spread of infectious diseases such as COVID-19 is an inherently spatial process. To assess the potential of spatial autocorrelation to bias our results, we calculated the daily unweighted average incidence of all bordering counties for each US county. This raw average of bordering counties’ incidence was used as a control in both AFT and GLM models as sensitivity analyses. We also ran spatial autoregressive models accounting for spatial autoregressive errors using the *spregress* command in STATA 15. We found that GLM models that adjusted for spatial autocorrelation led to similar findings to the models included in this paper.

## 3 Results

Table 1, Table 2 show the results of the AFT models examining the association between past county-level NHH spending in Table 1 — or state-level spending in Table 2— and time elapsed between the 10th case and incidence curve bending. Model 1 includes only the county (state) level spending variables which relate to our primary hypothesis. In Model 2, socio-demographic covariates and testing rates are added to Model 1. Similarly, Model 3 incorporates additional covariates capturing income to Model 2; Model 4 adds covariates that capture health policy and population health variables; and Model 5 adds temperature; and political preferences in terms of % of 2016 Republican votes at the county level are added in Model 6. In Table 1, we find no statistically significant association between pre-COVID county level NHH spending and more rapid control of COVID-19 incidence in terms of time to peak. We also do not find a statistically significant association between county level hospital spending and rapid control of COVID-19. These findings held across the simplest as well as all other model specifications. Although county level revenue per capita was associated with a shorter time to peak in Models 1 and 2, this association disappears as we include control variables in Models 3 to 6. We checked the robustness of our findings by restricting the time period of analysis to the first 90, 120, and 150 days of the pandemic (in addition to the main results that restrict the analysis to the first 180 days), and find that the lack of statistical association between county level per capita NHH spending persists across these alternative time periods.

In Table 2, we find that logged state level spending per capita on HPR is associated with a 30% shorter time to peak (Odds Ratio = 0.674 in Model 6) and therefore faster control over COVID-19 in the first wave across all specifications. State level spending on HPR involves making plans that involve public communication resources, law enforcement, school systems, and emergency services. This is precisely the part of state PH spending that would be expected to help improve the execution of contact tracing, testing, and compliance with lock down and quarantine in response to new challenges. We find that the state PH spending per capita on HPR remains significant across model specifications when we restrict the period for analysis to the first 120, and 150 days from the beginning of the pandemic. However, we find state level PH spending on HPR was not significant in models that restrict the time period of analysis to the first 90 days of the pandemic (results not shown in tables). The communicable disease budgets of many states are often devoted to specific reportable diseases like STDs, tuberculosis, rabies, etc., and although those capabilities were applicable to COVID-19, however, our analysis did not find a statistically significant effect of CoDC spending levels to time to achieve COVID-19 control.

Table 3, Table 4 show results from GLM with logged DT in the first 30 days of the epidemic as the dependent variable. Models 1–6 in these specifications incorporate additional covariates in a similar block-wise fashion as the time to event models. We note that county level NHH spending is associated with a shorter logged DT consistently across all model specifications in Table 3. Further, while pre-COVID per capita total state level spending increased DT in Models 2–6, we find that pre-COVID state level PH spending allocations to HPR and CoDC were not associated with DT consistently across model specifications in Table 4.

**Table 3:** Effect of county-level spending - Estimated coefficients from generalized linear models for log doubling time of incidence rates in first 30 days among counties that started an epidemic.

	Spending only	Spending + Testing + Demographic	Spending + Testing + Demographic + Income	Spending + Testing + Demographic + Income + Health	Spending + Testing + Demographic + Income + Health + Temperature	Spending + Testing + Demographic + Income + Health + Temperature + Political
	(1)	(2)	(3)	(4)	(5)	(6)
	Model 1	Model 2	Model 3	Model 4	Model 5	Model 6
Ln(County Non-Hospital Health Spending Per Capita)	-0.018*	-0.021**	-0.021**	-0.020**	-0.019**	-0.016*
	[0.010]	[0.009]	[0.009]	[0.009]	[0.009]	[0.009]
Ln(County Revenue Per Capita)	0.047	-0.012	-0.031	-0.058	-0.072	-0.057
	[0.049]	[0.045]	[0.045]	[0.046]	[0.046]	[0.046]
Ln(County Hospital Spending Per Capita)	-0.009**	-0.011***	-0.008**	-0.006	-0.005	-0.006
	[0.004]	[0.003]	[0.003]	[0.004]	[0.004]	[0.004]
Ln(County Public Welfare Spending Per Capita)	-0.014*	-0.003	-0.010	-0.015**	-0.013*	-0.012*
	[0.008]	[0.007]	[0.007]	[0.007]	[0.007]	[0.007]

Observations	2,029	2,029	2,029	2,029	2,029	2,029

Standard errors in brackets. Spending coefficients correspond to natural log of 1 + actual spending to avoid taking log of zero.

***p < 0.01, **p < 0.05, *p < 0.1.

**Table 4:** Effect of State Level Spending - Estimated coefficients from generalized linear models for log doubling time of incidence rates in first 30 days among counties that started an epidemic.

	Spending only	Spending + Testing + Demographic	Spending + Testing + Demographic + Income	Spending + Testing + Demographic + Income + Health	Spending + Testing + Demographic + Income + Health + Temperature	Spending + Testing + Demographic + Income + Health + Temperature + Political
	(1)	(2)	(3)	(4)	(5)	(6)
	Model 1	Model 2	Model 3	Model 4	Model 5	Model 6
Ln(State Per Capita Spending - Total)	0.022	0.072***	0.063***	0.064***	0.048**	0.045**
	[0.022]	[0.021]	[0.021]	[0.022]	[0.022]	[0.022]
Ln(State Per Capita Spending – Hazard Preparation and Response)	-0.010	0.012	0.007	-0.003	-0.025	-0.020
	[0.028]	[0.024]	[0.024]	[0.025]	[0.025]	[0.025]
Ln(State Per Capita Spending - Communicable Disease Control)	-0.046***	-0.021*	-0.017	-0.020*	-0.011	-0.007
	[0.013]	[0.011]	[0.011]	[0.012]	[0.012]	[0.012]

Observations	1,992	1,992	1,992	1,992	1,992	1,992

Standard errors in brackets. Spending coefficients correspond to natural log of 1 + actual spending to avoid taking log of zero.

***p < 0.01, **p < 0.05, *p < 0.1.

Although the models are not designed explicitly to look at the impact of covariates, we note that across all models estimated in Table 1, Table 2 (complete tables are available in supplementary appendices), higher logged state testing rates, logged percent Hispanic, percent African American, percent of population below the age of 18, percent of population above the age of 65, logged ratio of males to females, and active primary care physicians per 100,000 were statistically significant and protective in that they were associated with shorter time to bend incidence curves. Conversely, a higher proportion of college graduates significantly lengthened the time to bend the curve. The logged density of population, while having odds ratios above 1, was not statistically significant across all models estimated in Table 1, Table 2 All statistically significant findings should be interpreted bearing in mind that most of the counties in the analysis were right censored because they had not bent their curve. In the models estimated in Table 3, Table 4 (complete tables are available in supplementary appendices), we note that higher logged population density, logged state testing rates, logged percent Hispanic, logged percent African American, and percent of the population that is obese shortened DT in the first 30 days of an epidemic.

## 4 Discussion and conclusion

States and counties in the US have displayed great heterogeneity in their ability to control the pandemic. Our research asks if county and state level governments in the US that spent more on NHH at the county level and PH at the state level prior to the pandemic were better prepared to manage the COVID-19 pandemic in the first half of 2020.

Our evidence shows that county level NHH expenditure prior to the pandemic was not associated with better COVID-19 control, at least at the outset of the COVID-19 pandemic. This finding held across multiple specifications of COVID-19 control at the county-level. However, there are tentative results showing that state-level PH spending on HPR may have played a role in controlling the pandemic among counties that were able to bend their curves. The results were statistically significant for HPR having a role in time to bend a COVID-19 curve, but not for doubling time. This finding may reflect the slow speed of the flow of state level resources to LHDs and inability to move resources (both financial and personnel) around to manage COVID-19 in the first 30 days of the pandemic. Further, given that pre-COVID spending variables are from before 2018, our results cannot unpack how state level PH spending was geographically allocated at the LHD and community levels to manage pandemics. This limitation in our study is shared by others as pointed out by Singh who notes —“how increased public health spending translates into improved population health outcomes remains a black box” (Singh, 2014).

Although not conclusive, our results suggest that being prepared to address PH challenges at the scale of the COVID-19 may require more than just marginal changes in funding for state and local PH agencies. Specifically, without structural changes in how funds are allocated and spent merely increasing resources at the state and local level during years when there is not a major crisis without attention to bolstering the foundational capacities in state and local health departments is unlikely to be sufficient to prepare the PH system for future epidemics and emergency preparedness.

American's local health budgets are fragmented into programmatic budget siloes. State and federal grantors hold local governments accountable for specific delivery of countable services. Local governments have very little discretionary spending and lack the ability to invest in core foundational capabilities, such as communications, community partnerships, epidemiological intelligence, or administrative capacity. Staff who have spent their careers in a specific categorical project may not be well-equipped to pivot to address emerging threats and challenges. From this perspective, it may not be so surprising that county level NHH or public hospital expenditures prior to the pandemic were not associated with better COVID-19 control across multiple specifications. Furthermore, because state resources are more abundant and in the case of HPR more closely configured for flexibility it is consistent to find that state-level spending on HPR may have played a role in controlling the pandemic among counties that were able to bend their curves. As guidance for future policies to improve local health department pandemic preparedness, our results offer no support for simply pouring more funds into the old fragmented and categorical system as a method to prepare for future outbreaks. More funding must be allied with support for the foundational capabilities of PH in assessment, policy development, and assurance executed via broad multi-sectoral partnerships involving every local health department.

The PH system in the US remains largely a series of earmarked categorical allocations to various disease conditions and special populations leaving little discretion at the local health levels for unfunded and unanticipated needs such as the COVID-19 pandemic. Although, there was substantial pre-existing variation in how NHH activities locally were funded prior to COVID-19, both well-funded and poorly funded counties appeared to be equally overwhelmed in their attempts to bend their local outbreak curves. Prior public funds spent the way they had been spent up until 2020 did not seem to offer advantages to health departments in slowing COVID-19. Our findings suggest that building the infrastructure to prepare for future outbreaks may require a more purposeful emphasis on investments in the foundational capabilities inside health departments (DeSalvo et al., 2021). Our results are unable to provide any insights into how PH spending ought to be restructured, but higher aggregated NHH spending at the county level alone does not appear to be protective in the management of COVID-19, which suggests that pouring more funds into the system without thoughtful attention to bolstering the foundational capabilities in state and local health departments may not better prepare the US PH system for future emergencies. A number of leaders in the field have called for future state and local PH funds to be directed more specifically to capabilities supporting the PH system itself (Castrucci et al., 2021; DeSalvo et al., 2021; National Network of Public Health Institutes, 2021).

### 4.1 Limitations

There were several limitations in this study. First, the historical NHH and PH spending data were from 2018 or earlier and required us to assume that counties and states did not have large alterations in their governmental health spending thereafter. Recently published research documents the stability of year-to-year spending on PH in US states and counties after 2008, suggesting that this assumption may be reasonable for the purposes of this study (Alfonso et al., 2021). However, there are many reports that by 2020 states and counties did activate emergency financing to help their LHDs respond to COVID-19, which affects the validity of pre-pandemic spending indicators as relevant determinants of a health department's resources during the late Spring and Summer of 2020. To cope with this limitation, we estimated models with DT in the first 30 days of the start of a county's local epidemic.

We are also limited by the US Census Bureau's definitions and categories for local government spending. The local government Census data captures only three major health related activities for local governments: reimbursements to vendors for medical services, public hospital spending, and all other non-hospital health spending. We use the latter two categories to compute measures of hospital and NHH, but we cannot determine where LHDs spent these funds. Similarly, a small number of the US Census Bureau's estimates for state level spending relate directly to health spending (Leider et al., 2018). At the state level, granular coding of the category of non-hospital spending could be used to disaggregate this spending category into HPR, CoDC, maternal and child health, among other categories (Resnick et al., 2017). We used the relevant categories of state PH spending on HPR and CoDC in this paper. Our finding that logged state level spending per capita on HPR is associated with a shorter time to peak suggests that the granularity of PH spending data is important – however this granularity is unavailable at the county level.,

Another limitation was that the main indicator of success in controlling COVID-19 (bending the incidence curve) was right censored in most US counties. Hence, most of what informed our time-to-event models was the timing of achieving success among successful counties, with unsuccessful counties all grouped together as being right censored. Repeating this analysis for the time period until 2021, when more of the counties will have hopefully bent the curve, would overcome this limitation, but introduce bias due to the non-random allocation of federal CARES Act funding. Analysis of COVID-19 data in late 2020 and into 2021 would be subject to the limitation that much more emergency PH funding arriving at counties would have rendered measures of pre-COVID PH capacity less and less relevant to the ability to control outbreaks. If the access to CARES ACT funding was an omitted variable that correlated positively with pre-pandemic spending the omitted variable bias ought to have magnified, rather than reduced the protective effect of pre-pandemic spending by counties. Since pre-pandemic spending had a null effect, the omitted variable bias must be negligible.

One limitation of our inclusion criteria could be that including only counties with 10 or more cases in the time frame of analysis excludes some small rural counties from the analysis. To address this limitation, we carried out a stratified analysis where counties were separated into rural and urban counties, and focused just on rural counties. We used the National Center for Health Statistics classification to identify rural counties (CDC). We found that the lack of association between county government health spending variables and speed of COVID-19 control was robust in models with only rural counties.

Lastly, we are aware that making causal claims will be challenging as it may not be possible to disentangle the effects of pre-existing social and political environments from local government health spending. Nevertheless, we did find that state level spending on HPR appeared to have been protective, which suggests that unobservable confounding and right censoring were not impediments to the detection of any correlations. The analysis used a large set of controls that were included in a block-wise fashion and accounted for spatial autocorrelation in incidence rates to overcome some of these issues.

### 4.2 Conclusion

This analysis looked specifically at associations between county-level control of COVID-19 and pre-COVID state PH and local non-hospital health funding. Our findings suggest that greater pre-pandemic county level health expenditures were not associated with better COVID-19 control. At least at the outset of the COVID-19 pandemic, having higher baseline spending did not automatically lead to better COVID-19 outcomes. Our results do not show that pre-COVID funding had no impact on human health as county health department budgets are allocated to a wide range of priorities beyond COVID-19. However, our results suggest that a county's preparedness to address PH challenges at the scale of COVID-19 may require more substantial investment in PH and population-based prevention, compared to other disease or program specific health-related spending areas that localities invest in. Structural changes to support basic PH foundational capabilities may be necessary to keep the PH system prepared for future epidemics.

## Author statement

All authors have seen and approved the final version of the manuscript that is being uploaded. The authors gratefully acknowledge the following student volunteers who contributed quality checks on the data: Julia Burleson, Collete Chang, Sharon Chow, Jake Griffin,Kyuhee Jo, Jamie Lee, Amanda Maldonado, Isabella Sarria, Caden Riley, Teagan Toomre, Samantha Walch, Cali Wilson.

## Funding

This research was supported by the Hopkins Population Center Grant (P2CHD042854) from the National Institute of Child Health and Human Development, and by the Robert Wood Johnson Foundation through its Systems for Action Research Program (grant 78116).

## Human participant compliance statement

This research is not human subjects research and did not need ethical approval as it uses publicly available ecological data at the US county and state level.

## Declaration of competing interest

The authors declare that they have no conflicts of interest.

## Footnotes

1 We use curve “bending” rather than curve “flattening” as a measure of COVID-19 control success because we believe that curve “bending” is more amenable to mathematical definition than “flattening”. “Bending” requires the second derivative to change from positive to negative, while “flattening” requires the second derivative to change from positive to zero. We do not observe incidence slopes that stay exactly equal to the 0 in the case incidence curves among counties in the period under consideration. A definition of “flattening” would involve arbitrary choices about how close to zero an incidence slope would need to be over what period of time to qualify as flattening. In theory, it is possible to have a mix of “natural history” counties that reached a natural peak quickly by letting the epidemic spread unchecked, as well as “flattened curve” counties that wrestled their epidemic to a long-drawn-out peak. In that case, the “fast peak” counties would be those with the worst public health performance. In practice, we know that none of the counties in the US reached their natural peaks in the first part of 2020.

2 DT can be calculated from fitting an exponential model Y=Y0Exp(qj t) to the case curve where Y is the case count, qj is the exponential parameter for the j-th county and t is time since the 10th case. The model requires assuming that the q parameter is relatively constant during the period studied. This assumption is only appropriate when the fraction of susceptibles is relatively large and when human efforts to control transmission have not yet led to large changes in q. DT can be calculated as log 2/q.
