# Supplementary material for: Past local government health spending was not correlated with COVID-19 control in US counties
Source: SSM Popul Health. 2022 Jan 18;17:101027. doi: 10.1016/j.ssmph.2022.101027 (PMC8763410; doi:10.1016/j.ssmph.2022.101027)
Supplement: Multimedia component 1 [file mmc1.docx]

**Supplementary Digital Content Tables and Figures**

Supplementary Table 1: Summary Statistics

| **Variable** | **Mean** | **SD** | **N** |
| --- | --- | --- | --- |
| Peak COVID-19 Incidence Rate per 100,000 | 17.55 | 18.37 | 2029 |
| Log of Peak Incident Cases per 100,000 as of July 19, 2020 | 2.43 | 0.96 | 2029 |
| Doubling time in first 30 days | 16.57 | 9.73 | 2029 |
| ***County level spending (and revenue)*** | | | |
| County Non-Hospital Health Spending Per Capita | 145.27 | 197.91 | 2029 |
| County Revenue Per Capita | 5393.29 | 1832.56 | 2029 |
| County Hospital Spending Per Capita | 522.98 | 947.85 | 2029 |
| County Public Welfare Spending Per Capita | 123.63 | 197.70 | 2029 |
| ***State level public health spending*** | | | |
| State Per Capita Spending - Total (2018 US$) | 151.71 | 111.98 | 1992 |
| State Per Capita Public Health Spending - Communicable Disease Control (CoDC) | 9.02 | 10.60 | 1992 |
| State Per Capita Public Health Spending - Hazard Preparation and Response (HPR) | 1.17 | 1.05 | 1992 |
| ***Controls: Demographic and Testing Rates*** | | | |
| Density (Population/Square Mile) | 168.49 | 332.46 | 2029 |
| COVID-19 testing rates per capita | 1.16 | 1.33 | 2029 |
| % of population that is Hispanic | 10.17 | 14.31 | 2029 |
| % of population that is Black/African American | 1.43 | 1.64 | 2029 |
| % of population that is under 18 years | 22.29 | 3.16 | 2029 |
| % of population that is over 65 | 18.63 | 4.15 | 2029 |
| % of population that is rural | 58.90 | 49.21 | 2029 |
| Net International Migrants as a % of Total Population | 0.00 | 0.00 | 2029 |
| Net Domestic Migrants as a % of Total Population | 0.01 | 0.01 | 2029 |
| Ratio of Males to Females | 1.00 | 0.10 | 2029 |
| ***Controls: Income*** | | | |
| % adults with at least some college education (2014-18) | 30.64 | 4.62 | 2029 |
| Income Inequality Ratio: 80th percentile to 20th percentile | 4.53 | 0.74 | 2029 |
| % of households that are food insecure | 13.41 | 4.38 | 2029 |
| Median household Income 2018 | 52736.46 | 12583.53 | 2029 |
| ***Controls: Health*** | | | |
| % of adults that are uninsured (2019) | 9.93 | 6.47 | 2029 |
| Active Primary Care Physicians per 100,000 Population 2018 (AAMC) | 87.19 | 12.34 | 2029 |
| % of adults that are current smokers (2020) | 16.63 | 3.73 | 2029 |
| % of adults that are Obese | 32.48 | 4.71 | 2029 |
| Age Adjusted Death Rate 100,000 (2016) | 825.58 | 133.77 | 2029 |
| ***Controls: Temperatures*** | | | |
| Average March Temperature (degrees Fahrenheit) | 42.80 | 11.46 | 2029 |
| Average April Temperature (degrees Fahrenheit) | 55.54 | 8.57 | 2029 |
| Average May Temperature (degrees Fahrenheit) | 64.37 | 9.43 | 2029 |
| ***Controls: Political*** | | | |
| % of 2020 Presidential votes for Republicans | 62.10 | 14.39 | 2029 |

Supplementary Table 2: Key Variables and Data Sources

| **Category** | **Key Variables** | **Data Sources** |
| --- | --- | --- |
| County Level Public Health Expenditure | - County Hospital Spending Per Capita - County Revenue Per Capita - County Non-Hospital Health Spending Per Capita - County Public Welfare Spending Per Capita | US Census Bureau [2,3,4,5,6,7]  Described in detail in McCullough and Leider (2016) |
| State Level Public Health Expenditure | - State Per Capita Spending - State Per Capita Public Health Spending - Communicable Disease Control (CoDC) - State Per Capita Public Health Spending - Hazard Preparation and Response (HPR) | Census Bureau’s State Finance Division [2,3,4,5,6,7]  Described in detail in Leider et al. 2016 |
| COVID-19 cumulative cases | - Peak Incidence Rate - Time to Peak Incidence Rate - Doubling Time of Incidence Rate | New York Times [1] |
| Demographic Indicators | - % of population below 18 - % of population above 65 - Sex Ratio - Density of Population/Sq. Mile - % Hispanic - % African-American | US Census Bureau [8] |
| Migration | - Net International Migrants as a % of Total Population - Net Domestic Migrants as a % of Total Population | Killeen et al. (2020) [12] |
| State Testing Rates | - State rates per capita (Positive, Negative, and Pending tests are considered) | COVID Tracking Project[12] |
| Temperatures | - Mean temperatures in March - Mean temperatures in April - Mean temperatures in May | Killeen et al. (2020) [11] |
| Population Health Outcomes | - % adults who smoked - % adults who are obese - Age adjusted all-cause mortality rates per 100,000 | County Health Rankings [9]  CDC Wonder Underlying Cause of Death [13] |
| Income | - Income Inequality Ratio: 80th pctl to 20th pctl - % households that are income insecure | County Health Rankings [9] |
| Education | - % adults with some college education | Killeen et al. (2020) [11] |
| Health Policy | - Active Primary Care Physicians per 100000 Population 2018 - % population uninsured | Killeen et al. (2020) [11] |
| Political Affiliations | - Proportion of Republican Votes at the county level for the 2016 Presidential Elections | MIT Election Data and Science Lab [10] |

Data Sources Referenced in Appendix Table 1.

[1] New York Times, <https://github.com/nytimes/covid-19-data> and CSSE, JHU. "COVID-19 Data Repository by the Center for Systems Science and Engineering (CSSE) at Johns Hopkins University." (2020).

[2] U.S. Census Bureau. Government Finance and Employment Classification Manual, Washington, DC (2006). Available from: [https://www2.census.gov/govs/pubs/classification/2006_classification_manual.pdf](https://nam02.safelinks.protection.outlook.com/?url=https%3A%2F%2Fwww2.census.gov%2Fgovs%2Fpubs%2Fclassification%2F2006_classification_manual.pdf&data=02%7C01%7Cslamba1%40jhu.edu%7C9602aa92475e4d085ec508d861733a7c%7C9fa4f438b1e6473b803f86f8aedf0dec%7C0%7C0%7C637366494809691339&sdata=8P%2B8PjAoxPsok84TolLj9xa4ADNqVt5HTjrodmyyfmc%3D&reserved=0)

[3] McCullough JM, Leider JP. Government spending in health and non-health sectors associated with improvement in county health rankings. Health Aff (Millwood). 2016;35(11):2037–43.

[4] Resnick BA, Fisher JS, Colrick IP, Leider JP. The Foundational Public Health Services as a Framework for Estimating Spending. American Journal of Preventive Medicine. 2017;53(5):646-51.

[5] Bekemeier B, Singh SR, Schoemann AW. A uniform chart of accounts for public health agencies: an “essential ingredient” for a strong public health system. Journal of Public Health Management and Practice. 2018;24(3):289-91.

[6] Leider J, Resnick B, Sensenig A, Alfonso Y, Brady E, Colrick I, et al. Assessing the Public Health Activity Estimate from the National Health Expenditure Accounts: Why Public Health Expenditure Definitions Matter. Journal of Health Care Finance. 2016;43(2).

[7] U.S. Bureau of Economic Analysis, Government consumption expenditures and gross investment: State and local (implicit price deflator) [A829RD3Q086SBEA], retrieved from FRED, Federal Reserve Bank of St. Louis; [https://fred.stlouisfed.org/series/A829RD3Q086SBEA](https://nam02.safelinks.protection.outlook.com/?url=https%3A%2F%2Ffred.stlouisfed.org%2Fseries%2FA829RD3Q086SBEA&data=02%7C01%7Cslamba1%40jhu.edu%7C9602aa92475e4d085ec508d861733a7c%7C9fa4f438b1e6473b803f86f8aedf0dec%7C0%7C0%7C637366494809701331&sdata=79HyMYMEgI3RoOKT%2BRTFPJ9%2F3Fcl7xp04pPyrOI7Po4%3D&reserved=0) [Accessed April 2020].

[8] U.S. Census Bureau. Intercensal Estimates of the Resident Population for the United States, Regions, States, and Puerto Rico. Available from: [https://www.census.gov/data/tables.html](https://nam02.safelinks.protection.outlook.com/?url=https%3A%2F%2Fwww.census.gov%2Fdata%2Ftables.html&data=02%7C01%7Cslamba1%40jhu.edu%7C9602aa92475e4d085ec508d861733a7c%7C9fa4f438b1e6473b803f86f8aedf0dec%7C0%7C0%7C637366494809701331&sdata=HAy8KVslVt7yfBmP3AF1b9W2vXZatqqEJJSIV0qiIAA%3D&reserved=0)   [Accessed July 2020].

[9]County Health Rankings and Roadmaps (2020): <https://www.countyhealthrankings.org/explore-health-rankings/measures-data-sources/2020-measures>

[10]MIT Election Data and Science Lab, 2018, "County Presidential Election Returns 2000-2016", https://doi.org/10.7910/DVN/VOQCHQ, Harvard Dataverse

[11]Killeen BD, Wu JY, Shah K, Zapaishchykova A, Nikutta P, Tamhane A, et al. A County-level Dataset for Informing the United States’ Response to COVID-19 [Inernet]. Baltimore (MD): Johns Hopkins University; 2020 [cited 2020 May 11]. Available from: <https://arxiv.org/pdf/2004.00756v1> .pdf

Complete dataset available from: <https://github.com/JieYingWu/COVID-19_US_County-level_Summaries/tree/master/data>

[12] COVID tracking project. https://covidtracking.com/.

[13] CDC Wonder Underlying Cause of Death Data. <http://wonder.cdc.gov/wonder/help/mcd.html>

Supplementary Table 3: Effect of county-level spending - Estimated odds ratios from AFT models with time to peak as dependent variable

|  | Spending only | Spending + Testing + Demographic | Spending + Testing + Demographic + Income | Spending + Testing + Demographic + Income + Health | Spending + Testing + Demographic + Income + Health + Temperature | Spending + Testing + Demographic + Income + Health + Temperature + Political |
| --- | --- | --- | --- | --- | --- | --- |
|  | (1) | (2) | (3) | (4) | (5) | (6) |
| VARIABLES | Model 1 | Model 2 | Model 3 | Model 4 | Model 5 | Model 6 |
|  |  |  |  |  |  |  |
| Ln(County Non-Hospital Health Spending Per Capita) | 1.043 | 0.992 | 0.990 | 0.993 | 0.993 | 0.993 |
|  | [0.0584] | [0.0574] | [0.0571] | [0.0569] | [0.0559] | [0.0561] |
| Ln(County Revenue Per Capita) | 0.449*** | 0.638* | 0.664 | 0.715 | 0.751 | 0.756 |
|  | [0.107] | [0.162] | [0.171] | [0.185] | [0.194] | [0.198] |
| Ln(County Hospital Spending Per Capita) | 0.983 | 0.986 | 0.986 | 0.981 | 0.980 | 0.980 |
|  | [0.0178] | [0.0186] | [0.0186] | [0.0187] | [0.0186] | [0.0187] |
| Ln(1 + County Public Welfare Spending Per Capita) | 0.983 | 0.988 | 0.992 | 1.002 | 1.017 | 1.018 |
|  | [0.0490] | [0.0509] | [0.0518] | [0.0511] | [0.0509] | [0.0511] |
| Ln(Density of Pop/SqMi) |  | 1.169** | 1.189** | 1.188** | 1.133 | 1.137 |
|  |  | [0.0900] | [0.0929] | [0.0923] | [0.0916] | [0.0939] |
| Ln(State Testing Rates) |  | 0.656*** | 0.652*** | 0.648*** | 0.664*** | 0.664*** |
|  |  | [0.0421] | [0.0421] | [0.0409] | [0.0412] | [0.0412] |
| Ln(% Hispanic) |  | 0.786** | 0.728*** | 0.726*** | 0.687*** | 0.689*** |
|  |  | [0.0762] | [0.0774] | [0.0778] | [0.0778] | [0.0794] |
| Ln(% African American) |  | 0.794** | 0.799** | 0.807** | 0.834* | 0.835* |
|  |  | [0.0809] | [0.0838] | [0.0860] | [0.0897] | [0.0900] |
| % under 18 years |  | 0.925*** | 0.916*** | 0.920** | 0.930** | 0.929** |
|  |  | [0.0277] | [0.0282] | [0.0304] | [0.0303] | [0.0305] |
| % over 65 |  | 0.932*** | 0.925*** | 0.923*** | 0.919*** | 0.918*** |
|  |  | [0.0218] | [0.0223] | [0.0228] | [0.0224] | [0.0227] |
| Rural |  | 0.998 | 0.998 | 0.998 | 0.998 | 0.998 |
|  |  | [0.00148] | [0.00153] | [0.00154] | [0.00155] | [0.00156] |
| Ln(Absolute Per Capita International Migration) |  | 0.971 | 0.981 | 0.971 | 0.992 | 0.991 |
|  |  | [0.0551] | [0.0562] | [0.0563] | [0.0578] | [0.0578] |
| Ln(Absolute Per Capita Domestic Migration) |  | 1.068 | 1.075 | 1.072 | 1.064 | 1.065 |
|  |  | [0.0497] | [0.0499] | [0.0501] | [0.0496] | [0.0497] |
| Ln(Ratio of Males to Females) |  | 0.0306*** | 0.0258*** | 0.0294*** | 0.0315*** | 0.0312*** |
|  |  | [0.0255] | [0.0217] | [0.0251] | [0.0269] | [0.0267] |
| % adults with some college educ (2014-18) |  | 1.053*** | 1.054*** | 1.052*** | 1.059*** | 1.059*** |
|  |  | [0.0166] | [0.0170] | [0.0180] | [0.0185] | [0.0187] |
| Ln(Median Household Income) |  |  | 0.749 | 0.618 | 0.607 | 0.608 |
|  |  |  | [0.340] | [0.324] | [0.320] | [0.321] |
| Income Inequality Ratio: 80th pctl to 20th pctl |  |  | 1.007 | 1.009 | 0.971 | 0.974 |
|  |  |  | [0.109] | [0.110] | [0.108] | [0.109] |
| % of households food insecure |  |  | 0.959* | 0.958* | 0.946** | 0.948** |
|  |  |  | [0.0227] | [0.0239] | [0.0239] | [0.0255] |
| % Uninsured Adults (2019) |  |  |  | 0.993 | 0.988 | 0.988 |
|  |  |  |  | [0.0227] | [0.0220] | [0.0220] |
| Active Primary Care Physicians per 100000 Population 2018 (AAMC) |  |  |  | 0.946*** | 0.955*** | 0.955*** |
|  |  |  |  | [0.0124] | [0.0121] | [0.0122] |
| % Adult Smoking (2020) |  |  |  | 0.982 | 0.984 | 0.984 |
|  |  |  |  | [0.0238] | [0.0236] | [0.0236] |
| % Obese Adult Population |  |  |  | 0.992 | 0.996 | 0.996 |
|  |  |  |  | [0.0164] | [0.0167] | [0.0167] |
| Age Adjusted Death Rate 100,000 (2016) |  |  |  | 1.000 | 1.000 | 1.000 |
|  |  |  |  | [0.000667] | [0.000665] | [0.000677] |
| Mar Temp AVG / F |  |  |  |  | 1.115*** | 1.115*** |
|  |  |  |  |  | [0.0379] | [0.0380] |
| Apr Temp AVG / F |  |  |  |  | 0.887** | 0.886** |
|  |  |  |  |  | [0.0522] | [0.0524] |
| May Temp AVG / F |  |  |  |  | 1.022 | 1.022 |
|  |  |  |  |  | [0.0448] | [0.0449] |
| % Voted for Republicans (2016) |  |  |  |  |  | 1.001 |
|  |  |  |  |  |  | [0.00646] |
| /ln_p | 0.953 | 0.942 | 0.941 | 0.935 | 0.937 | 0.937 |
|  | [0.0420] | [0.0419] | [0.0422] | [0.0427] | [0.0407] | [0.0411] |
| /lntheta | 1.479 | 2.594** | 3.110** | 1.363 | 0.845 | 0.845 |
|  | [0.590] | [1.195] | [1.486] | [0.531] | [0.349] | [0.349] |
| Constant | 182,134*** | 25,188*** | 1.063e+06** | 1.674e+09*** | 1.829e+09*** | 1.577e+09*** |
|  | [364,725] | [62,451] | [6.118e+06] | [1.125e+10] | [1.248e+10] | [1.084e+10] |
|  |  |  |  |  |  |  |
| Observations | 147,346 | 147,346 | 147,346 | 147,346 | 147,346 | 147,346 |
| Number of groups | 48 | 48 | 48 | 48 | 48 | 48 |
| AIC | 2846 | 2721 | 2723 | 2718 | 2712 | 2714 |
| Subjects | 1957 | 1957 | 1957 | 1957 | 1957 | 1957 |
| Time at Risk | 151676 | 151676 | 151676 | 151676 | 151676 | 151676 |
| seEform in brackets |  |  |  |  |  |  |
| *** p<0.01, ** p<0.05, * p<0.1 |  |  |  |  |  |  |

Supplementary Table 4 Effect of state-level spending - Estimated odds ratios from AFT models with time to peak as dependent variable

|  | Spending only | Spending + Testing + Demographic | Spending + Testing + Demographic + Income | Spending + Testing + Demographic + Income + Health | Spending + Testing + Demographic + Income + Health + Temperature | Spending + Testing + Demographic + Income + Health + Temperature + Political |
| --- | --- | --- | --- | --- | --- | --- |
|  | (1) | (2) | (3) | (4) | (5) | (6) |
| VARIABLES | Model 1 | Model 2 | Model 3 | Model 4 | Model 5 | Model 6 |
|  |  |  |  |  |  |  |
| Ln(State per capita spending - Total) | 0.701 | 0.716 | 0.721 | 0.917 | 0.915 | 0.911 |
|  | [0.155] | [0.176] | [0.185] | [0.216] | [0.192] | [0.192] |
| Ln(1 + State Per Capita Spending - Hazard Prep) | 0.593** | 0.517*** | 0.512*** | 0.542*** | 0.600** | 0.600** |
|  | [0.136] | [0.120] | [0.123] | [0.121] | [0.122] | [0.122] |
| Ln(1 + State Per Capita Spending - Communicable Disease Control) | 1.042 | 0.973 | 0.978 | 1.018 | 0.934 | 0.936 |
|  | [0.149] | [0.164] | [0.173] | [0.149] | [0.119] | [0.119] |
| Ln(Density of Pop/SqMi) |  | 1.191** | 1.206** | 1.214** | 1.153* | 1.158* |
|  |  | [0.0902] | [0.0925] | [0.0930] | [0.0917] | [0.0940] |
| Ln(State Testing Rates) |  | 0.663*** | 0.660*** | 0.654*** | 0.674*** | 0.674*** |
|  |  | [0.0422] | [0.0424] | [0.0409] | [0.0413] | [0.0413] |
| Ln(% Hispanic) |  | 0.749*** | 0.696*** | 0.681*** | 0.659*** | 0.663*** |
|  |  | [0.0723] | [0.0736] | [0.0725] | [0.0740] | [0.0760] |
| Ln(% African American) |  | 0.781** | 0.786** | 0.792** | 0.816* | 0.818* |
|  |  | [0.0804] | [0.0837] | [0.0855] | [0.0886] | [0.0890] |
| % under 18 years |  | 0.925*** | 0.914*** | 0.913*** | 0.920** | 0.919** |
|  |  | [0.0277] | [0.0281] | [0.0305] | [0.0303] | [0.0304] |
| % over 65 |  | 0.932*** | 0.923*** | 0.921*** | 0.917*** | 0.916*** |
|  |  | [0.0217] | [0.0222] | [0.0228] | [0.0225] | [0.0227] |
| Rural |  | 0.997* | 0.998 | 0.998 | 0.998 | 0.998 |
|  |  | [0.00146] | [0.00152] | [0.00153] | [0.00154] | [0.00155] |
| Ln(Absolute Per Capita International Migration) |  | 0.978 | 0.991 | 0.982 | 0.998 | 0.997 |
|  |  | [0.0562] | [0.0576] | [0.0576] | [0.0585] | [0.0584] |
| Ln(Absolute Per Capita Domestic Migration) |  | 1.063 | 1.068 | 1.067 | 1.062 | 1.062 |
|  |  | [0.0499] | [0.0499] | [0.0502] | [0.0497] | [0.0498] |
| Ln(Ratio of Males to Females) |  | 0.0356*** | 0.0290*** | 0.0316*** | 0.0327*** | 0.0322*** |
|  |  | [0.0298] | [0.0244] | [0.0272] | [0.0279] | [0.0275] |
| % adults with some college educ (2014-18) |  | 1.053*** | 1.053*** | 1.054*** | 1.062*** | 1.061*** |
|  |  | [0.0166] | [0.0171] | [0.0181] | [0.0188] | [0.0189] |
| Ln(Median Household Income) |  |  | 0.751 | 0.612 | 0.612 | 0.616 |
|  |  |  | [0.343] | [0.322] | [0.325] | [0.326] |
| Income Inequality Ratio: 80th pctl to 20th pctl |  |  | 0.976 | 0.976 | 0.941 | 0.946 |
|  |  |  | [0.106] | [0.107] | [0.105] | [0.106] |
| % of households food insecure |  |  | 0.959* | 0.958* | 0.948** | 0.950* |
|  |  |  | [0.0231] | [0.0242] | [0.0242] | [0.0258] |
| % Uninsured Adults (2019) |  |  |  | 1.002 | 1.000 | 1.000 |
|  |  |  |  | [0.0232] | [0.0225] | [0.0225] |
| Active Primary Care Physicians per 100000 Population 2018 (AAMC) |  |  |  | 0.949*** | 0.958*** | 0.959*** |
|  |  |  |  | [0.0124] | [0.0123] | [0.0125] |
| % Adult Smoking (2020) |  |  |  | 0.981 | 0.982 | 0.982 |
|  |  |  |  | [0.0240] | [0.0237] | [0.0238] |
| % Obese Adult Population |  |  |  | 0.993 | 0.994 | 0.994 |
|  |  |  |  | [0.0165] | [0.0168] | [0.0168] |
| Age Adjusted Death Rate 100,000 (2016) |  |  |  | 1.000 | 1.000 | 1.000 |
|  |  |  |  | [0.000672] | [0.000669] | [0.000679] |
| Mar Temp AVG / F |  |  |  |  | 1.105*** | 1.106*** |
|  |  |  |  |  | [0.0376] | [0.0376] |
| Apr Temp AVG / F |  |  |  |  | 0.882** | 0.882** |
|  |  |  |  |  | [0.0528] | [0.0529] |
| May Temp AVG / F |  |  |  |  | 1.039 | 1.039 |
|  |  |  |  |  | [0.0475] | [0.0475] |
| % Voted for Republicans (2016) |  |  |  |  |  | 1.002 |
|  |  |  |  |  |  | [0.00637] |
| /ln_p | 0.943 | 0.937 | 0.937 | 0.930* | 0.934 | 0.934 |
|  | [0.0415] | [0.0423] | [0.0422] | [0.0409] | [0.0422] | [0.0418] |
| /lntheta | 1.227 | 1.826 | 2.184* | 1.074 | 0.679 | 0.678 |
|  | [0.433] | [0.745] | [0.934] | [0.403] | [0.269] | [0.269] |
| Constant | 1,651*** | 5,146*** | 373,484** | 2.731e+08*** | 2.750e+08*** | 2.308e+08*** |
|  | [1,959] | [9,688] | [2.118e+06] | [1.806e+09] | [1.842e+09] | [1.550e+09] |
|  |  |  |  |  |  |  |
| Observations | 143,877 | 143,877 | 143,877 | 143,877 | 143,877 | 143,877 |
| Number of groups | 47 | 47 | 47 | 47 | 47 | 47 |
| AIC | 2831 | 2693 | 2695 | 2689 | 2683 | 2685 |
| Subjects | 1919 | 1919 | 1919 | 1919 | 1919 | 1919 |
| Time at Risk | 148132 | 148132 | 148132 | 148132 | 148132 | 148132 |
| seEform in brackets |  |  |  |  |  |  |
| *** p<0.01, ** p<0.05, * p<0.1 |  |  |  |  |  |  |

Supplementary Table 5: Effect of county-level spending - Estimated coefficients from generalized linear models for log doubling time of incidence rates in first 30 days among counties that started an epidemic

|  | Spending only | Spending + Testing + Demographic | Spending + Testing + Demographic + Income | Spending + Testing + Demographic + Income + Health | Spending + Testing + Demographic + Income + Health + Temperature | Spending + Testing + Demographic + Income + Health + Temperature + Political |
| --- | --- | --- | --- | --- | --- | --- |
|  | (1) | (2) | (3) | (4) | (5) | (6) |
| VARIABLES | Model 1 | Model 2 | Model 3 | Model 4 | Model 5 | Model 6 |
|  |  |  |  |  |  |  |
| Ln(County Non-Hospital Health Spending Per Capita) | -0.018* | -0.021** | -0.021** | -0.020** | -0.019** | -0.016* |
|  | [0.010] | [0.009] | [0.009] | [0.009] | [0.009] | [0.009] |
| Ln(County Revenue Per Capita) | 0.047 | -0.012 | -0.031 | -0.058 | -0.072 | -0.057 |
|  | [0.049] | [0.045] | [0.045] | [0.046] | [0.046] | [0.046] |
| Ln(County Hospital Spending Per Capita) | -0.009** | -0.011*** | -0.008** | -0.006 | -0.005 | -0.006 |
|  | [0.004] | [0.003] | [0.003] | [0.004] | [0.004] | [0.004] |
| Ln(1 + County Public Welfare Spending Per Capita) | -0.014* | -0.003 | -0.010 | -0.015** | -0.013* | -0.012* |
|  | [0.008] | [0.007] | [0.007] | [0.007] | [0.007] | [0.007] |
| Ln(Density of Pop/SqMi) |  | -0.114*** | -0.125*** | -0.117*** | -0.096*** | -0.091*** |
|  |  | [0.014] | [0.014] | [0.014] | [0.016] | [0.016] |
| Ln(State Testing Rates) |  | -0.028*** | -0.027*** | -0.020** | -0.021*** | -0.016** |
|  |  | [0.008] | [0.008] | [0.008] | [0.008] | [0.008] |
| Ln(% Hispanic) |  | -0.113*** | -0.112*** | -0.106*** | -0.135*** | -0.125*** |
|  |  | [0.015] | [0.016] | [0.018] | [0.021] | [0.021] |
| Ln(% African American) |  | -0.072*** | -0.084*** | -0.104*** | -0.116*** | -0.109*** |
|  |  | [0.023] | [0.023] | [0.024] | [0.024] | [0.024] |
| % under 18 years |  | 0.001 | -0.008 | -0.003 | -0.009 | -0.012* |
|  |  | [0.006] | [0.007] | [0.007] | [0.007] | [0.007] |
| % over 65 |  | 0.012** | 0.006 | 0.005 | 0.002 | -0.000 |
|  |  | [0.005] | [0.005] | [0.005] | [0.005] | [0.005] |
| % rural population |  | 0.001*** | 0.001*** | 0.001*** | 0.001*** | 0.001*** |
|  |  | [0.000] | [0.000] | [0.000] | [0.000] | [0.000] |
| Ln(Absolute Per Capita International Migration) |  | 0.026** | 0.025** | 0.025** | 0.024** | 0.026** |
|  |  | [0.011] | [0.011] | [0.011] | [0.011] | [0.011] |
| Ln(Absolute Per Capita Domestic Migration) |  | -0.016 | -0.012 | -0.010 | -0.010 | -0.008 |
|  |  | [0.010] | [0.010] | [0.010] | [0.010] | [0.010] |
| Ln(Ratio of Males to Females) |  | 0.277* | 0.018 | 0.046 | -0.055 | -0.152 |
|  |  | [0.163] | [0.177] | [0.178] | [0.176] | [0.182] |
| % adults with some college educ (2014-18) |  | 0.009*** | 0.007** | 0.007** | 0.004 | 0.004 |
|  |  | [0.003] | [0.003] | [0.003] | [0.003] | [0.003] |
| Ln(Median Household Income) |  |  | 0.063 | 0.010 | -0.064 | -0.069 |
|  |  |  | [0.097] | [0.109] | [0.109] | [0.110] |
| Income Inequality Ratio: 80th pctl to 20th pctl |  |  | -0.013 | -0.017 | -0.016 | -0.009 |
|  |  |  | [0.022] | [0.022] | [0.022] | [0.022] |
| % of households food insecure |  |  | -0.012*** | -0.008* | -0.008 | -0.002 |
|  |  |  | [0.004] | [0.005] | [0.005] | [0.005] |
| % Uninsured Adults (2019) |  |  |  | -0.001 | 0.001 | 0.001 |
|  |  |  |  | [0.003] | [0.003] | [0.003] |
| Active Primary Care Physicians per 100000 Population 2018 (AAMC) |  |  |  | 0.003** | 0.004*** | 0.005*** |
|  |  |  |  | [0.001] | [0.001] | [0.001] |
| % Adult Smoking (2020) |  |  |  | -0.001 | -0.000 | -0.001 |
|  |  |  |  | [0.004] | [0.004] | [0.004] |
| % Obese Adult Population |  |  |  | -0.009*** | -0.009*** | -0.009*** |
|  |  |  |  | [0.003] | [0.003] | [0.003] |
| Age Adjusted Death Rate 100,000 (2016) |  |  |  | 0.000 | 0.000 | -0.000 |
|  |  |  |  | [0.000] | [0.000] | [0.000] |
| Mar Temp AVG / F |  |  |  |  | -0.012** | -0.011** |
|  |  |  |  |  | [0.005] | [0.005] |
| Apr Temp AVG / F |  |  |  |  | 0.051*** | 0.048*** |
|  |  |  |  |  | [0.008] | [0.008] |
| May Temp AVG / F |  |  |  |  | -0.035*** | -0.035*** |
|  |  |  |  |  | [0.006] | [0.006] |
| % Voted for Republicans (2016) |  |  |  |  |  | 0.004*** |
|  |  |  |  |  |  | [0.001] |
| Constant | 2.565*** | 3.182*** | 3.333*** | 3.964*** | 4.935*** | 4.713*** |
|  | [0.401] | [0.434] | [1.165] | [1.357] | [1.366] | [1.369] |
|  |  |  |  |  |  |  |
| Observations | 2,029 | 2,029 | 2,029 | 2,029 | 2,029 | 2,029 |
| Standard errors in brackets |  |  |  |  |  |  |
| *** p<0.01, ** p<0.05, * p<0.1 |  |  |  |  |  |  |

Supplementary Table 6: Effect of State Level Spending - Estimated coefficients from generalized linear models for log doubling time of incidence rates in first 30 days among counties that started an epidemic

|  | Spending only | Spending + Testing + Demographic | Spending + Testing + Demographic + Income | Spending + Testing + Demographic + Income + Health | Spending + Testing + Demographic + Income + Health + Temperature | Spending + Testing + Demographic + Income + Health + Temperature + Political |
| --- | --- | --- | --- | --- | --- | --- |
|  | (1) | (2) | (3) | (4) | (5) | (6) |
| VARIABLES | Model 1 | Model 2 | Model 3 | Model 4 | Model 5 | Model 6 |
|  |  |  |  |  |  |  |
| Ln(State per capita spending - Total) | 0.022 | 0.072*** | 0.063*** | 0.064*** | 0.048** | 0.045** |
|  | [0.022] | [0.021] | [0.021] | [0.022] | [0.022] | [0.022] |
| Ln(1 + State Per Capita Spending - Hazard Prep) | -0.010 | 0.012 | 0.007 | -0.003 | -0.025 | -0.020 |
|  | [0.028] | [0.024] | [0.024] | [0.025] | [0.025] | [0.025] |
| Ln(1 + State Per Capita Spending - Communicable Disease Control) | -0.046*** | -0.021* | -0.017 | -0.020* | -0.011 | -0.007 |
|  | [0.013] | [0.011] | [0.011] | [0.012] | [0.012] | [0.012] |
| Ln(Density of Pop/SqMi) |  | -0.114*** | -0.125*** | -0.116*** | -0.098*** | -0.092*** |
|  |  | [0.013] | [0.013] | [0.014] | [0.016] | [0.016] |
| Ln(State Testing Rates) |  | -0.015* | -0.015* | -0.009 | -0.014* | -0.010 |
|  |  | [0.008] | [0.008] | [0.008] | [0.008] | [0.008] |
| Ln(% Hispanic) |  | -0.109*** | -0.111*** | -0.117*** | -0.148*** | -0.135*** |
|  |  | [0.016] | [0.016] | [0.018] | [0.021] | [0.021] |
| Ln(% African American) |  | -0.096*** | -0.105*** | -0.118*** | -0.124*** | -0.116*** |
|  |  | [0.023] | [0.023] | [0.024] | [0.025] | [0.025] |
| % under 18 years |  | -0.001 | -0.009 | -0.006 | -0.010 | -0.012* |
|  |  | [0.007] | [0.007] | [0.007] | [0.007] | [0.007] |
| % over 65 |  | 0.009* | 0.002 | 0.002 | -0.001 | -0.003 |
|  |  | [0.005] | [0.005] | [0.005] | [0.005] | [0.005] |
| % rural population |  | 0.001*** | 0.001*** | 0.001*** | 0.001*** | 0.001*** |
|  |  | [0.000] | [0.000] | [0.000] | [0.000] | [0.000] |
| Ln(Absolute Per Capita International Migration) |  | 0.027** | 0.026** | 0.025** | 0.023** | 0.026** |
|  |  | [0.011] | [0.011] | [0.011] | [0.011] | [0.011] |
| Ln(Absolute Per Capita Domestic Migration) |  | -0.010 | -0.004 | -0.004 | -0.006 | -0.004 |
|  |  | [0.010] | [0.011] | [0.011] | [0.011] | [0.011] |
| Ln(Ratio of Males to Females) |  | 0.221 | -0.045 | -0.012 | -0.075 | -0.160 |
|  |  | [0.167] | [0.183] | [0.183] | [0.181] | [0.185] |
| % adults with some college educ (2014-18) |  | 0.007*** | 0.004 | 0.004 | 0.003 | 0.003 |
|  |  | [0.003] | [0.003] | [0.003] | [0.003] | [0.003] |
| Ln(Median Household Income) |  |  | 0.008 | -0.037 | -0.099 | -0.098 |
|  |  |  | [0.100] | [0.112] | [0.111] | [0.112] |
| Income Inequality Ratio: 80th pctile to 20th pctile |  |  | -0.015 | -0.017 | -0.019 | -0.011 |
|  |  |  | [0.022] | [0.022] | [0.022] | [0.022] |
| % of households food insecure |  |  | -0.012*** | -0.010** | -0.009* | -0.003 |
|  |  |  | [0.004] | [0.005] | [0.005] | [0.006] |
| % Uninsured Adults (2019) |  |  |  | -0.000 | 0.001 | 0.001 |
|  |  |  |  | [0.003] | [0.003] | [0.003] |
| Active Primary Care Physicians per 100000 Population 2018 (AAMC) |  |  |  | 0.001 | 0.003* | 0.003** |
|  |  |  |  | [0.001] | [0.001] | [0.001] |
| % Adult Smoking (2020) |  |  |  | -0.001 | -0.001 | -0.001 |
|  |  |  |  | [0.005] | [0.005] | [0.004] |
| % Obese Adult Population |  |  |  | -0.011*** | -0.011*** | -0.010*** |
|  |  |  |  | [0.003] | [0.003] | [0.003] |
| Age Adjusted Death Rate 100,000 (2016) |  |  |  | 0.000 | 0.000 | 0.000 |
|  |  |  |  | [0.000] | [0.000] | [0.000] |
| Mar Temp AVG / F |  |  |  |  | -0.011** | -0.011** |
|  |  |  |  |  | [0.005] | [0.005] |
| Apr Temp AVG / F |  |  |  |  | 0.050*** | 0.047*** |
|  |  |  |  |  | [0.009] | [0.009] |
| May Temp AVG / F |  |  |  |  | -0.033*** | -0.032*** |
|  |  |  |  |  | [0.007] | [0.007] |
| % Voted for Republicans (2016) |  |  |  |  |  | 0.004*** |
|  |  |  |  |  |  | [0.001] |
| Constant | 2.797*** | 2.841*** | 3.476*** | 4.027*** | 4.599*** | 4.407*** |
|  | [0.114] | [0.296] | [1.185] | [1.379] | [1.384] | [1.391] |
|  |  |  |  |  |  |  |
| Observations | 1,992 | 1,992 | 1,992 | 1,992 | 1,992 | 1,992 |
| Standard errors in brackets |  |  |  |  |  |  |
| *** p<0.01, ** p<0.05, * p<0.1 |  |  |  |  |  |  |
